# Diagnosing Lung Abnormalities Related to Heart Failure in Chest Radiogram, Lung Ultrasound and Thoracic Computed Tomography

**DOI:** 10.3390/arm91020010

**Published:** 2023-02-23

**Authors:** Dominika Siwik, Wojciech Apanasiewicz, Małgorzata Żukowska, Grzegorz Jaczewski, Marta Dąbrowska

**Affiliations:** 1 Department of Internal Medicine, Pulmonary Diseases and Allergy, Medical University of Warsaw, 02-091 Warsaw, Poland; 2 Students’ Research Group ‘Alveolus’, Department of Internal Medicine, Pulmonary Diseases and Allergy, Medical University of Warsaw, 02-091 Warsaw, Poland; 3 2nd Department of Clinical Radiology, Medical University of Warsaw, Banacha 1A, 02-097 Warsaw, Poland

**Keywords:** heart failure, chest CT, lung ultrasound, chest X-ray, imaging

## Abstract

**Highlights:**

**What are the main findings?**

- Various imaging techniques are useful in differentiating lung abnormalities related to HF from pulmonary diseases.
- Lung ultrasound is a novel and emerging imaging tool for the management of HF.

**What is the implication of the main finding?**

- Recognising lung abnormalities in heart failure is crucial in routine clinical diagnostics.

**Abstract:**

Heart failure (HF) is a multidisciplinary disease affecting almost 1–2% of the adult population worldwide. Symptoms most frequently reported by patients suffering from HF include dyspnoea, cough or exercise intolerance, which is equally often observed in many pulmonary diseases. The spectrum of lung changes related to HF is wide. The knowledge of different types of these abnormalities is essential to distinguish patients with HF from patients with lung diseases or both disorders and thus avoid unnecessary diagnostics or therapies. In this review, we aimed to summarise recent research concerning the spectrum of lung abnormalities related to HF in three frequently used lung imaging techniques: chest X-ray (CXR), lung ultrasound (LUS) and chest computed tomography (CT). We discussed the most prevalent abnormalities in the above-mentioned investigations in the context of consecutive pathophysiological stages identified in HF: (i) redistribution, (ii) interstitial oedema, and (iii) alveolar oedema. Finally, we compared the utility of these imaging tools in the clinical setting. In conclusion, we consider LUS the most useful and promising imaging technique due to its high sensitivity, repeatability and accessibility. However, the value of CXR and chest CT is their potential for establishing a differential diagnosis.

## 1. Introduction

Heart failure (HF) is a widely prevalent disease that affects around 65 million people worldwide [1]. Solely in the USA, HF-associated expenditure is predicted to double from 2012 to 2030 [2]. Costs are not associated with hospitalisations alone but also result from the need to maintain the optimal quality of life by alleviating everyday symptoms. These include fatigue, dyspnoea, exercise intolerance or oedema of the lower extremities. The presence of these symptoms and objective evidence of heart dysfunction are essential for the diagnosis of HF. Recently, the significance of lung ultrasound (LUS) as an additional diagnostic method has been increasing. However, echocardiography (ECHO) is still the key diagnostic tool to assess the type of HF. Based on the measurement of ejection fraction (EF) in ECHO, HF may present as three phenotypes: (1) heart failure with reduced systolic function (EF ≤ 40%; HFrEF), (2) with mildly reduced systolic function (EF 41–49%; HFmrEF) and (3) with preserved systolic function (EF ≥ 50%; HFpEF). The diagnosis of HFmrEF or HFrEF is based on symptoms and results of ECHO, while to establish the diagnosis of HFpEF, an additional criterion needs to be met—either elevated serum BNP or evidence of cardiac structural or functional abnormalities (left ventricular (LV) diastolic dysfunction or raised LV filling pressures) documented in ECHO or other imaging tests such as cardiac magnetic resonance, single-photon emission computed tomography or positron emission tomography [3].

According to recent studies, the group of patients with HFpEF is increasing most rapidly [4,5]. This is an important issue as precise diagnosis of HFpEF is still challenging, while the prognosis in this group remains poor [6]. Moreover, patients with HFpEF differ from other subjects, with HF being older and presenting with more comorbidities [7]. The similarity of signs and symptoms in certain conditions other than cardiovascular, e.g., anaemia, pulmonary or renal disease, to signs and symptoms of HF may lead to misdiagnosis and inappropriate management [7]. Thus, it is important to precisely diagnose patients with HF and distinguish them from patients with other diseases, including diseases of the lung. Nowadays, imaging techniques such as chest X-ray (CXR), chest computed tomography (CT) or lung ultrasonography (LUS) play a key role in diagnosing the majority of lung disorders. They are also useful in assessing patients with HF, but on the other hand, they may be misleading as HF (particularly HFrEF) impacts the lungs and may cause some abnormalities in their imaging [8].

The knowledge of the interaction between the heart and lungs is essential in the understanding and interpretation of lung abnormalities in subjects with HF. Therefore, this review aimed to (1) outline the wide spectrum of lung abnormalities related to HF in the three most common lung imaging techniques: CXR, LUS and chest CT; (2) explain the pathomechanism responsible for the sequence of lung changes related to HF; and finally (3) analyse the advantages and disadvantages of these three imaging methods.

## 2. Chest X-ray

CXR is one of the most commonly available imaging tests used in the emergency department, especially in patients with dyspnoea, which frequently detect pulmonary disorders. Still, it may also demonstrate cardiac abnormalities [9,10]. Sometimes it can be useful as the first-step imaging test to distinguish acute heart failure (AHF) from acute lung disease [11]. Despite its absence in the main diagnostic pathway of chronic heart failure (CHF), CXR remains a recommended additional test [7]. In addition to its diagnostic role, CXR may also have a prognostic value. HF signs in CXR are associated with worse clinical outcomes for patients suffering from HF [12]. Llorens et al. showed that alveolar oedema found in CXR resulted in an 89% (95%CI 30–177%) higher in-hospital death rate and a 38% higher 1-year mortality [13].

Historically left and right heart failure was distinguished, with the first being more prevalent. The impact of left heart failure (HFpEF and HFrEF) on the lungs often leads to lung congestion, while right heart failure leads to systemic congestion expressed as distension of the jugular veins, peripheral oedema and organ dysfunction; thus, pulmonary symptoms are more obscure. However, right and left heart failure may coexist; in such cases, right HF is an important indicator of poor prognosis [14,15]. Moreover, right heart failure may also be a consequence of lung disease (e.g., COPD, interstitial lung disease (ILD), pulmonary embolism or pericardial diseases), which may impact imaging of the lungs.

The values of CXR are accessibility, low cost and low radiation risk (an estimated effective dose for CXR in posteroanterior view is 0.05 mSv and 0.10 mSv for posteroanterior and lateral projection) [16]. Therefore, CXR was applied as the first-step tool in the diagnosis of HF for many years, mostly in the acute setting. However, CXR is not deprived of limitations. Firstly, its diagnostic value in diagnosing HF is relatively low. A systematic review emphasised its moderate to high specificity (76–83%) but lower sensitivity (67–68%) in HF diagnosis [17]. The relatively low sensitivity of CXR in diagnosing HF results from the fact that radiographic signs of pulmonary oedema and vascular redistribution may be missed in radiogram as they depend on the patient’s position (recumbent vs. erect) and limited visualisation of retrocardiac opacities in PA radiogram. Additionally, the increased lymphatic flow may transiently balance the increase of hydrostatic pressure, thus preventing progress to pulmonary oedema visible in CXR [18,19,20]. Secondly, gravity has an impact on the results of CXR. Changing the position of the patient from orthostatic to supine may disturb the fluid flow resulting in a misleading impression of the patient’s condition [21]. Thus, the position of the patient should be taken into account in the comparison of the lobar vasculature. To sum up, CXR currently seems to be an insufficient imaging technique, and when HF is suspected, the need for further studies, including ECHO or LUS, is indisputable.

### 2.1. Pulmonary Features of HF in CXR

There are some elements in the pulmonary picture that are strongly connected with HF. The most spectacular is pulmonary oedema, a hallmark feature of AHF. Studies show that it is present in 28.7% of patients admitted to the hospital for AHF [22]. In addition, more than half of patients presenting with CHF will require hospitalisation for pulmonary oedema at least once in a lifetime [23]. 

**Figure 1:**
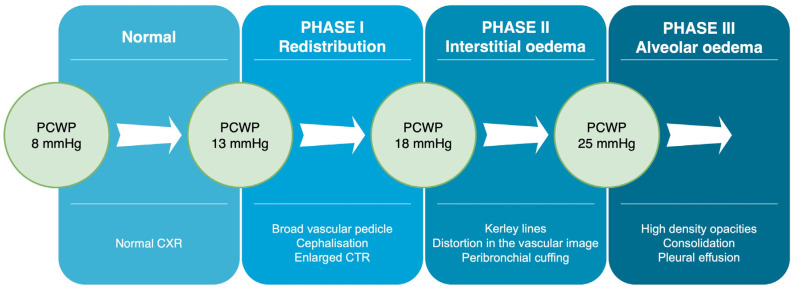
Consecutive phases of HF according to the pulmonary capillary wedge pressure (PCWP). Various pulmonary findings in CXR have been mentioned. CTR—Cardiac thoracic ratio; CXR—Chest X-ray.

From the pathophysiological point of view, the pathway to developing pulmonary oedema consists of three stages depending on increasing pulmonary capillary wedge pressure (Figure 1). It begins when the disruption of cardiac function increases hydrostatic pressure and redistribution in the lung vasculature (Phase 1). The overflow of lung vasculature results in fluid accumulation in the interstitium and the interlobular septa forming the first stage—interstitial oedema (Phase 2). When this space is overloaded, and lymphatics become congested, the excess fluid begins to accumulate in the alveoli, thus progressing to the subsequent stage—alveolar oedema (Phase 3) [18].

#### 2.1.1. Phase 1—Redistribution in the Lung Vasculature

**Figure 2:**
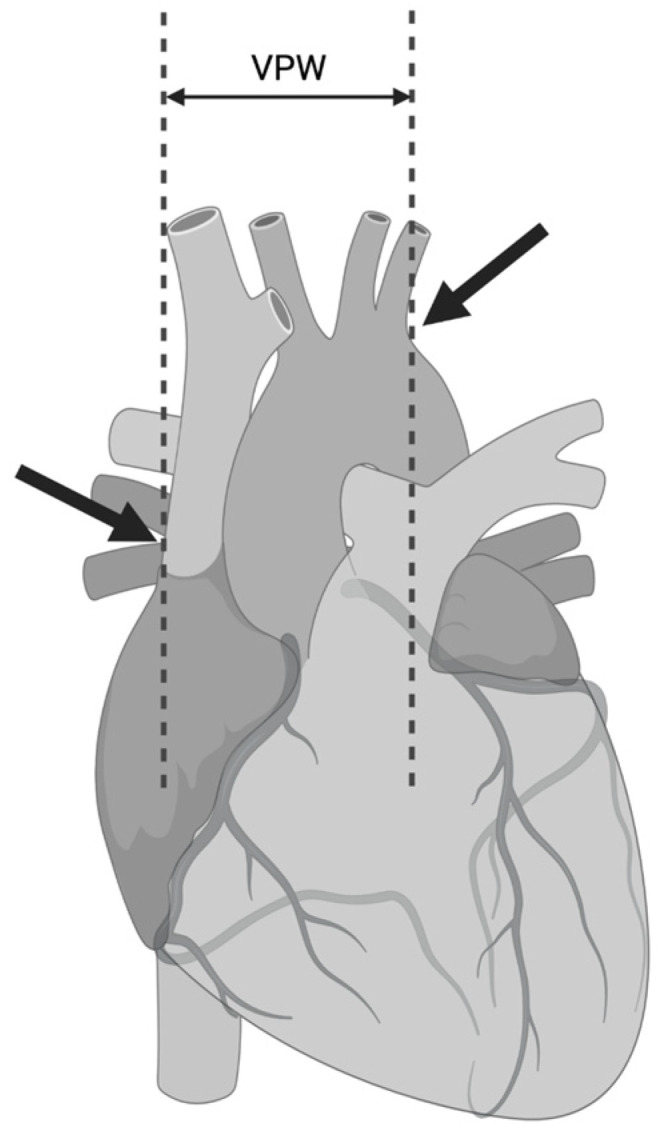
Vascular pedicle width. (Created with BioRender.com).

At first, cardiac failure leads to fluid redistribution and increased hydrostatic pressure in the large veins and the lung vessels. The process has its consequences in CXR images. To begin with, the first noticeable sign is the enlarged vascular pedicle width. It is assessed by measuring the distance between the two virtual vertical parallel lines on the CXR. The first line runs through the point at which the superior vena cava crosses the right main bronchus, while the second line passes through the beginning of the left subclavian artery [24] (Figure 2). The normal range of the perpendicular width should be less than about 48 ± 5 mm in the erect position with about a 20% increase in the supine [25]. The meta-analysis of 8 studies on 363 participants suggested that vascular pedicle width correlates with volume overload, which may help in the management of patients with HF [26].

The next feature of fluid redistribution as a consequence of HF visible in CXR are prominent pulmonary hila and enlarged veins of the upper lobes, known as cephalisation [27]. Under normal conditions, veins of the upper lobes have a smaller diameter than veins in the lower lobes due to gravity, but in HF, upper lobe vessels increase in size. According to the study by Mueller-Lenke et al., cephalisation had a sensitivity of 19.6% and specificity of 92.6% in recognising HF, respectively [28]. It is a subtle sign, present when the image is taken in an upright position and lost when the patient is in the supine position [29].

#### 2.1.2. Phase 2—Interstitial Oedema

The subsequent consequence of HF is interstitial oedema, which in CXR is reflected by: (1) distortion in the hilar image, (2) peribronchovascular cuffing (colloquially—“donut sign”), (3) thickening of interlobar or interlobular fissures [21]. The last feature is also known as the Kerley lines. The three types of Kerley lines are distinguished: A, B and C [30]. Kerley A lines represent the thickening of connections between the central and peripheral lymphatic systems. They are the longest Kerley lines spanning about 2 to 6 cm from the hilum to the upper lobes [31]. The second type—the B lines, are the most common finding in HF. They are short, 1 to 2 cm long, perpendicular to the pleura, emerging from the surface of the inferolateral areas of the lung [29]. Should the interstitial oedema incidents repeat, the interlobular fissures may undergo fibrosis, thus creating the so-called chronic Kerley B lines [32]. From the above-mentioned signs representing interstitial oedema, Kerley B lines are the most prominent in both values—sensitivity (23.4%) and specificity (95.8%) [28]. The third type—Kerley C lines, are the least frequent. These lines differ from the other Kerley lines in that they neither spread from the hilum nor reach the pleura and are the smallest in length.

#### 2.1.3. Phase 3—Alveolar Oedema

If excess fluid accumulates within the alveolar space, pulmonary oedema may manifest as alveolar oedema. Typically, its appearance on CXR is in the form of mostly bilateral higher density opacities in central and basal lung areas [27], what is referred to as the angel wing, bat wing or butterfly pattern [32]. The bat wing sign develops only in about 10% of pulmonary oedema cases. Furthermore, the sign may be asymmetrical, e.g., when the radiograph was taken while the patient was in a lateral position. It occurs mainly in the course of AHF, progressing so rapidly that it overloads the compensation mechanisms and covers the presence of interstitial oedema [33]. The rate of filtering of the excess fluid through the capillary wall in AHF may cause significant functional changes before morphological abnormalities appear on CXR. This explains why clinical symptoms tend to develop before radiological findings can be seen. The same applies to the resolution of the radiological findings—they resolve at a slower rate than clinical symptoms [33]. This may also explain the limited sensitivity of chest X-rays in patients with acute decompensated HF admitted to the ED [34].

#### 2.1.4. Phase 3—Pleural and/or Pericardial Effusion

Lastly, HF may manifest as pleural or pericardial effusion. In the retrospective analysis by Korczyński et al., CHF was the most common aetiology in patients with pleural effusion, accounting for 37.4% of all cases [35]. Interestingly, compared to patients with malignant and parapneumonic pleural effusion, HF-related pleural effusion was characterised by a lower fluid volume. In another retrospective review, 46% of 3245 patients diagnosed with acute decompensated HF presented signs of pleural effusion. In the analysed group, pleural effusion was bilateral in 58%, right-sided in 27%, and left-sided in 14% of cases, respectively [36]. In most patients with pleural effusion due to HF, pleural fluid is classified as transudate according to the Light criteria. However, 25% of cases may fall into the exudative category, especially if a patient is treated intensively with diuretics. In such cases, to differentiate whether the fluid is of cardiac origin, it is beneficial to assess the level of pleural NT-proBNP. Porcel et al. suggest that a pleural fluid BNP concentration exceeding 1500 pg/mL supports cardiac aetiology [37]. In addition, Morales-Rull et al. confirmed the viability of measuring NT-proBNP in serum while adding the parameters such as systolic pulmonary artery pressure and serum prealbumin in predicting PE development [36].

On the other hand, pericardial effusion, a rare symptom of HF, is associated with an elevated filling pressure of the right heart. It usually coexists with other signs of advanced congestive HF. As the frequency of left-sided overload dominates over the right-sided, pericardial effusion occurs less frequently than pleural fluid accumulation [38]. In the study conducted by Kataoka et al., from the 60 enrolled patients, 52 (87%) presented with pleural effusion, while only 12 (20%) had pericardial effusion, which was small to moderate in volume. No significant correlation between the pleural and pericardial effusion was found in that study [38]. Although pericardial effusion is rarely observed in HF it is associated with an unfavourable outcome, even if it is haemodynamically irrelevant [39,40,41]. In addition, in critically ill patients with relevant pericardial effusion treated in the Intensive Care Unit (ICU), a positive impact of pleural effusion drainage was documented [42].

**Figure 3:**
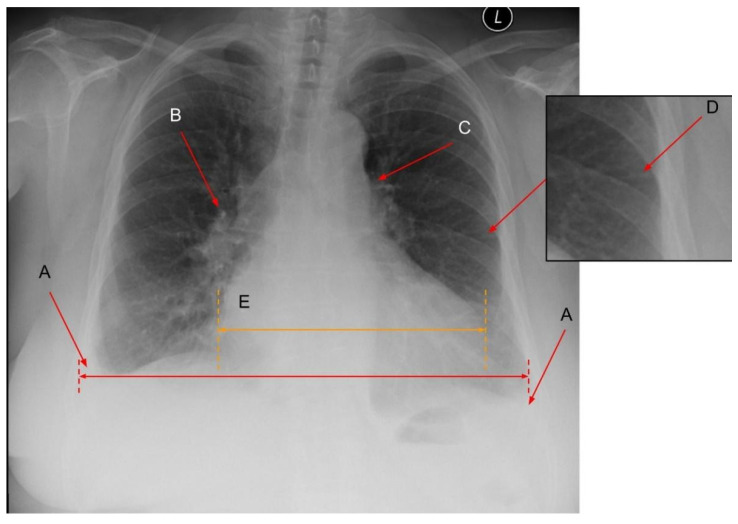
Pulmonary findings in a patient with HF. A: bilateral pleural effusion; B: increased diameter of the pulmonary vessels and the hazy contours, enlarged intermediate artery; C: Prominent pulmonary trunk; D: Interstitial oedema (Kerley B lines); E: Increased CTR > 0.5. B and C may signify the presence of pulmonary hypertension. Figure obtained with permission from the 2nd Department of Radiology, Medical University of Warsaw.

The spectrum of the above-mentioned pulmonary and heart findings related to HF visible in CXR is shown in Figure 3.

### 2.2. Cardiac Abnormalities Related to HF in CXR

**Figure 4:**
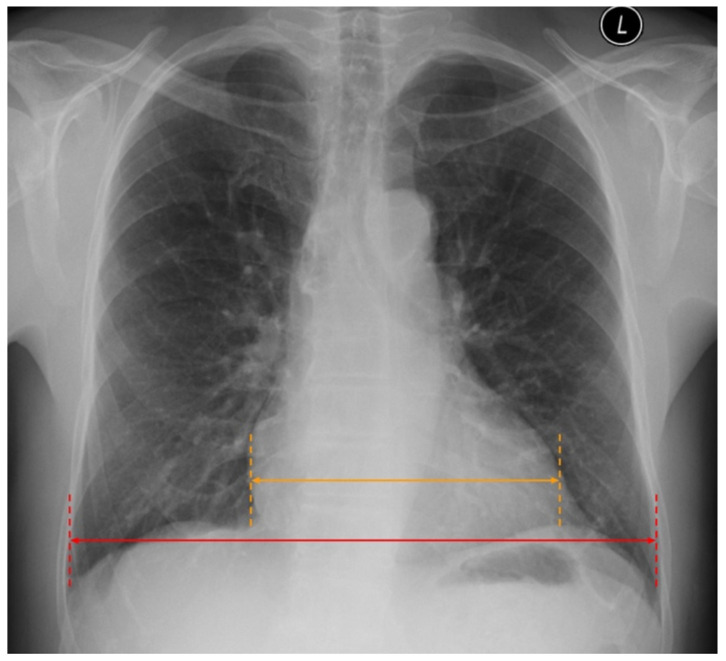
Increased cardiac thoracic ratio > 0.5 in CXR. Figure obtained with permission from the 2nd Department of Radiology, Medical University of Warsaw.

The most prominent abnormality detectable in CXR in patients with HF is cardiomegaly. It is simply defined as the subjectively enlarged heart. However, in radiological images, it is most often presented in the form of the ratio between the width of the heart and the thoracic cage, called the cardiac thoracic ratio (CTR). If the CTR is higher than 0.5—cardiomegaly might be suspected (Figure 4). However, it is worth mentioning that the dimensions of the heart and vessels are influenced by how the CXR is performed. Routinely CXR is taken in the PA (posterior-anterior) projection, which means that the course of the X-ray comes from the back of the patient. However, when CXR is taken in the AP (anterior-posterior) projection, the silhouette of the heart and vessels is enlarged. Thus, it is essential to report the projection applied.

In the study by Knudsen et al., cardiomegaly was the most frequent feature of HF in the radiographic, being present in 50% of affected patients [43]. Similar results were found in research by Fonesca et al., who observed that subjectively enlarged heart silhouette and CTR > 0.5 were present in CXR in 54% and 43% of subjects with HF, respectively [44]. The results of another study also confirmed that cardiomegaly was the most sensitive radiographic finding in congestive HF diagnosis [28]. However, the sensitivity of CXR in diagnosing cardiomegaly is rather limited. In the study by McKee et al., in a series of 244 patients with NSTEMI, which compared the accuracy of diagnosing cardiomegaly by CXR and echocardiography as the gold standard, the sensitivity of CXR to identify cardiomegaly was only 40%, specificity was 91%, positive and negative predictive values reached 56% and 84%, respectively. These findings show that CXR itself is insufficient for the diagnosis of HF and emphasise the need for implementing other diagnostic modalities, such as echocardiography [45]. In HFpEF, cardiomegaly is rarely present in the CXR. However, the left atrial enlargement was described in the form of splaying of the carina [46].

In conclusion, there is a considerable number of radiographic features of HF, which can be identified in CXR, and their correct radiological interpretation is of the essence. These features are specific but only moderately sensitive; thus, a chest radiogram as the only test is insufficient to confirm HF diagnosis [7]. However, it is helpful in the diagnosis of many other diseases (e.g., pneumonia, interstitial lung diseases, pneumothorax). Thus, it is an important first-step imaging test in diagnosing dyspnoeic patients, but other tests are usually necessary [7]. Recently, LUS has become an increasingly valuable tool for diagnosing patients with dyspnoea, particularly in the ED and ICU setting.

## 3. Lung Ultrasound (LUS)

Ultrasound is a relatively new but increasingly recognised technique in lung imaging, particularly useful in dyspnoeic or critically ill patients treated either in the ED or ICU [47]. In HF, LUS is primarily useful in finding pulmonary congestion, commonly found in HF patients admitted to ED. For that reason, LUS has earned its place in the new HF guidelines published by the European Society of Cardiology (ESC) in 2021. According to these guidelines, LUS may be considered in the diagnostic work-up of a suspected case of AHF [7]. It also enhances the establishment of a differential diagnosis. Furthermore, ultrasound imaging is low-cost, accurate, fast and available at the bedside. This article focuses on B-lines and pleural effusion, the hallmark signs of pulmonary congestion.

### 3.1. B-Lines

**Figure 5:**
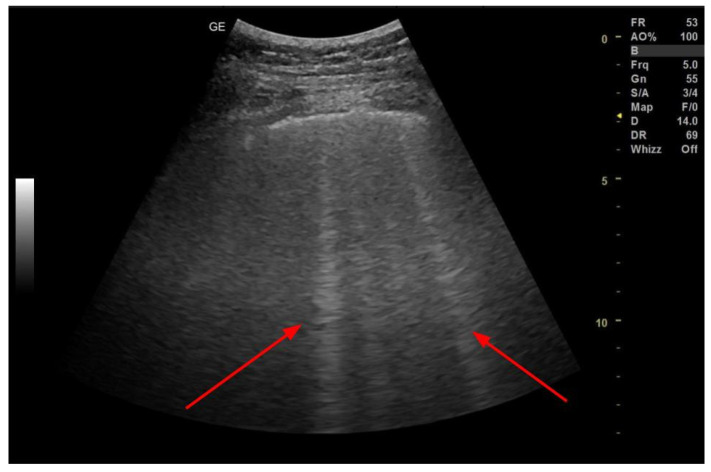
B-lines in LUS (Convex probe). Figure acquired in the Department of Internal Medicine, Pulmonary Diseases and Allergy, Medical University of Warsaw.

**Figure 6:**
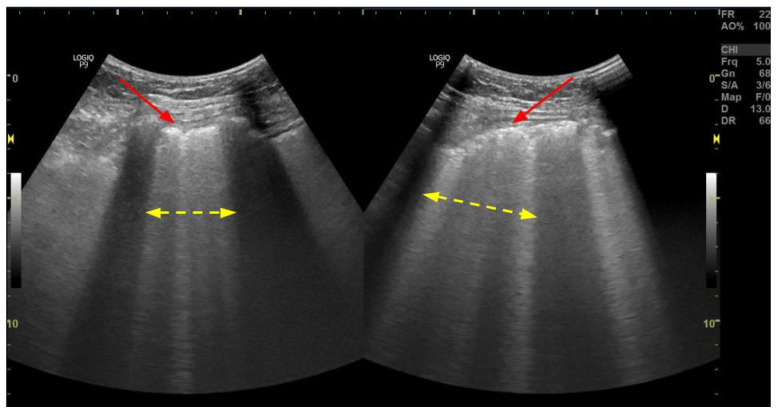
Ultrasonographic interstitial syndrome—more than 2 B-lines per intercostal space (yellow arrows) (“lung rockets”). Additionally, thickening and blurring of the pleural line and small subpleural consolidations are seen (red arrows). Figure acquired in the Department of Internal Medicine, Pulmonary Diseases and Allergy, Medical University of Warsaw.

B-lines, also called “lung comets” or “comet-tail artifacts,” are hyperechoic, laser-like vertical artefacts emerging from the pleural line, appearing through the whole screen of an LUS scan without fading [48] (Figure 5 and Figure 6). Although the exact mechanism leading to B-lines appearing in a sonogram is not fully understood, their presence and number are considered a measure of extravascular lung water (EVLW) volume [43]. B-lines are not seen in a fully aerated lung. They appear with EVLW volume increase—as in pulmonary congestion due to HF. It is believed that B-lines represent the thickening of the subpleural interstitial septae between pulmonary lobules and separate acini. As such, their number and spacing can be used to assess the severity of the pulmonary vasculature overload (i.e., the more severe the congestion, the more B-lines appear). Visualisation of up to two B-lines within one intercostal space in a single probe position is considered non-pathologic. The presence of three or more B-lines in one intercostal space is a defining feature of the sonographic interstitial syndrome—an artefactual image of the above-mentioned thickening of the interstitium. With increasing congestion, B-lines appear more and more abundantly, up to the point where it is impossible to distinguish and count them separately. This image, sometimes called “sonographic white lung”, defines the interstitial-alveolar syndrome—in the HF context, a manifestation of alveolar oedema. Further loss of lung aeration would result in the emergence of lung consolidations. In the course of HF, B-lines appear bilaterally, symmetrically, in gravity-dependent areas and tend to be homogeneously distributed. It is important to note these features, as B-lines alone are not specific for HF and, in fact, can be seen in the majority of lung pathologies as well. B-line distribution, presence or absence of other abnormalities in LUS image, and clinical context allow accurate interpretation of interstitial syndrome [48].

Many studies have shown that B-lines correlate not only with clinical outcomes but also with laboratory measurements such as serum BNP. For example, in the systematic review conducted by Muniz et al. analysing 26 articles, it was underlined that more than 15 B-lines, accounted for in all 28 chest zones, corresponded to serum BNP values higher than 500 pg/mL [49]. In addition, collective integration of both parameters (BNP and B-lines) may be beneficial in identifying the final diagnosis of the patient’s causes for congestion: haemodynamic or pulmonary [50].

Lung ultrasound continues to develop rapidly, and different methods are proposed to quantify B-lines efficiently [51]. Investigators in various studies used as few as 4 scans of the “wet spots” to as many as 28 scans [52,53]. A software-assisted approach was also described, and modern ultrasound systems sometimes offer functions designed to count B-lines [54]. In our experience in the hospital management of decompensated HF, semi-quantitative, simple assessment is sufficient to provide adequate care, while more complex, quantitative approaches tend to be too time-consuming and have little impact on treatment outcomes. In our routine practice, we use B-line number ranges and monitor their distribution and the number of intercostal spaces in which a significant number of B-lines (3+) is detectable. Consecutive studies in monitoring decompensated HF treatment should be performed using the same method with the patient in the same position and, if possible, by the same examiner.

As stated previously, CHF with repeating or persistent interstitial oedema may result in fibrosis of the peripheral elements of the lung interstitium. This may manifest in ultrasound as persistent B-lines and/or changes of the pleural line—thickening, irregularity or blurring.

On the one hand, it has been shown that more than three B-lines in the outpatient setting increase the risk for hospitalisation due to HF and all-cause mortality, even in the absence of signs in lung auscultation [55]. On the other hand, various studies suggest that the more B-lines are found in LUS, the worse the state patients present after discharge from the hospital. For instance, in the aforementioned meta-analysis, it has been found that less than 15 and 30 B-lines in LUS at discharge predicted longer event-free survival in 6- and 3-month follow-ups, respectively [49].

One of the crucial advantages of ultrasound is the possibility to dynamically assess the changes in the course of the disease [56]. In the meta-analysis by Maw et al., it has been proposed that the ultrasonographic signs of pulmonary oedema transition may be detected earlier than in CXR [19]. As other studies confirm, this can be beneficial in monitoring and modulating the diuretic treatment in emergency department patients, which in turn contributes to quicker discharge time [57]. It is worth noting that diuretic therapy in prehospital settings results in lower sensitivity in LUS in comparison to no previous treatment (57.73% vs. 83%) without significant changes in specificity (87.97% vs. 86.3%) [58].

There is insufficient data on the usefulness of LUS in differentiating HFpEF and HFrEF. While analysing these two HF subgroups, recent studies show no difference in pulmonary congestion measured in the number of B-lines [59,60]. However, the available studies underline that regular LUS in the outpatient setting is considered a prognostic factor of all-cause death and hospitalisation due to HF, independent of NT-proBNP measurement [61].

### 3.2. Inferior Vena Cava Measurement

In the course of HF, due to right ventricle dysfunction, distention of systemic veins may occur. Measurement of the inferior vena cava (IVC) diameter and its collapsibility on inspiration is rapid and an easy-to-perform method of preemptive assessment of right-heart failure [62,63,64]. IVC can be easily visualised through the acoustic window provided by liver parenchyma. Measurements can be obtained in both B- and M-modes, and the difference in diameter on inspiration and expiration should be noted. The examination is usually performed in a semi-recumbent position, in the longitudinal plane, with measurements being made 2 cm below the entry of IVC into the right atrium. Expiratory diameter exceeding 19–20 mm, inspiratory diameter exceeding 9–10 mm, and collapsibility on inspiration lower than approximately 50% are indicative of cardiac rather than pulmonary aetiology of acute dyspnoea in patients reporting to ED [62].

### 3.3. Pleural Effusion

**Figure 7:**
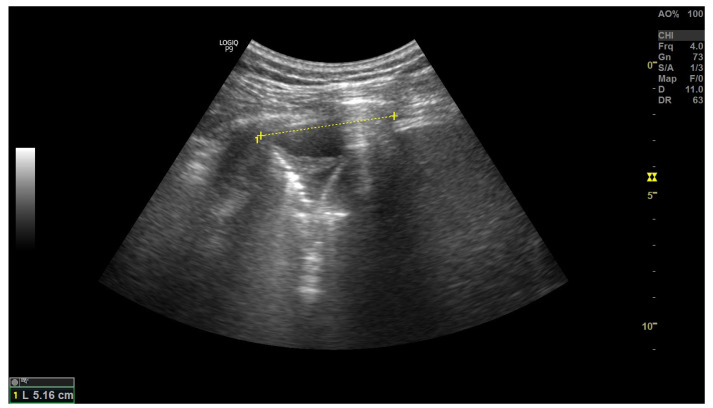
A very small amount of deeply hypoechoic free fluid in the costophrenic recess. Height of the fluid collection is measured. A small area of compression atelectasis. Figure acquired in the Department of Internal Medicine, Pulmonary Diseases and Allergy, Medical University of Warsaw.

**Figure 8:**
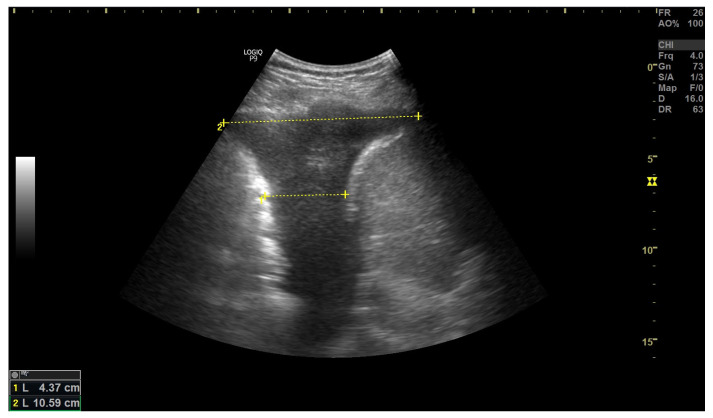
A small amount of simple fluid in the pleural cavity. Height of the effusion and distance between the lung base and diaphragm are measured. Figure acquired in the Department of Internal Medicine, Pulmonary Diseases and Allergy, Medical University of Warsaw.

Transthoracic ultrasound is a well-established method of investigating the pathology of the pleura. It is an excellent tool in diagnosing pleural effusion, considered by many a gold standard in this indication. The fluid detection limit is described as 5–20 mL, and for effusions exceeding 100 mL, ultrasound has been shown to be infallible [65]. Accumulating free fluid in the pleural cavity is seen as a separation of visceral and parietal pleura by a hypoechoic area. It is earliest detectable in the lowest posterolateral part of the costophrenic recess (Figure 7 and Figure 8). Usually, the presence of fluid in the pleural cavity results in the development of compression atelectasis, easily detectable by ultrasound.

Among many possible aetiologies of pleural effusion, HF is the most prevalent [35]. In the course of HF, it is usually bilateral if it develops unilaterally—twice more often on the right side. This is because the amount of free fluid in the pleural cavity is usually small to moderate.

As mentioned earlier, pleural effusion in the course of HF is usually free-flowing and transudative in nature. While it remains impossible to determine the nature and aetiology of pleural effusion based on ultrasound appearance alone, some characteristics may speak strongly against or for the diagnosis of a transudate. Transudative effusions contain few cells and little protein, as the underlying pathophysiology is hemodynamic rather than inflammatory. This usually results in a homogenic, anechoic or deeply hypoechoic appearance, with no structured echoes like strands of fibrin, septae or organised adhesions detectable within, no swirling sign present (swirling movement of small, dotty hyperechoic echoes within the fluid) and no thickening of parietal or diaphragmatic pleura. Ultrasound images of free fluid change shape when changing a patient’s position.

A plethora of methods to quantitatively determine the volume of pleural effusion using ultrasound have been proposed, all having limitations [66]. However, in everyday practice determining the amount of fluid semiquantitatively is usually sufficient for monitoring and adjusting treatment and establishing indications for diagnostic or therapeutic intervention (i.e., thoracentesis). Effusion volume may be described as trace, small, moderate, large or very large. Alternatively, the number of intercostal spaces in which effusion is detectable or how high the collection of fluid reaches in a sitting position may be given. When monitoring treatment, it is important to perform consecutive examinations with the patient in the same position.

The ability to provide qualitative and semi-quantitative characteristics of the pleural effusion makes transthoracic ultrasound an invaluable modality in the management of pleural effusions. It should be emphasized, however, that no ultrasound feature allows differentiating transudate and exudate with 100% specificity [67].

In summary, transthoracic ultrasound is an excellent diagnostic tool used in the management of HF. Its usage is gaining popularity due to emerging new evidence, ease of use and growing availability [68]. By visualising the above-mentioned B-lines, LUS plays an important role in pulmonary oedema detection. Moreover, LUS is more sensitive than radiography in diagnosing pleural or pericardial effusion and is also very useful in detecting lung consolidations [52,69]. Not only is it a promising instrument in ED, but it also provides great prognostic knowledge on CHF patients. Furthermore, ultrasound is not limited to pulmonary studies—in skilled hands; it may be used to comprehensively scan the lungs and quickly assess the condition of the heart or even perform a full echocardiographic study. However, because of the nature of ultrasonic waves, LUS has its limitations, which cannot be omitted. These, among others, include difficulties in performing the examination in obese patients and the lack of specificity in B-lines presence [70].

## 4. Chest CT

**Figure 9:**
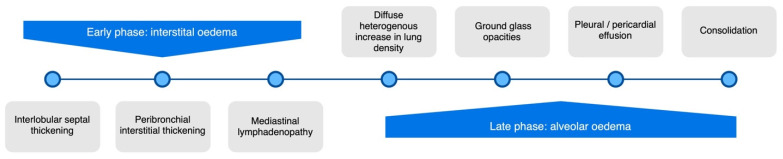
The following stages of pulmonary abnormalities related to HF in chest CT. More information in the text.

Chest CT is not commonly used to evaluate patients presenting with symptoms of acute and chronic HF. Symptoms and signs, blood tests and CXR are usually sufficient to establish the diagnosis of HF. However, CXR has limited accuracy in diagnosing different conditions, including HF. Although the diagnostic accuracy of chest CT in diagnosing lung diseases is higher than CXR, CT scanning has its limitations related to its costs, availability in emergencies and relatively high radiation exposure. However, patients with CHF may have other indications for chest CT, e.g., suspected lung cancer, ILD, bronchiectasis or pulmonary embolism. In such a situation, the diagnosis of pulmonary congestion is incidental. Furthermore, as HF impacts the lungs, some features related to HF may be found in the lungs and mediastinum in chest CT (Figure 9). Therefore, it may lead to diagnostic pitfalls and unnecessary diagnostic procedures. In this context, knowledge about the radiologic features of HF in chest CT may be useful.

**Figure 10:**
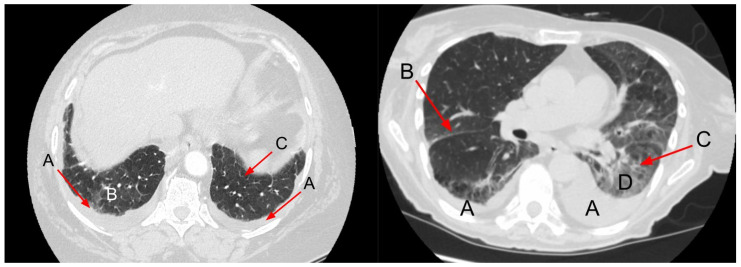
(**left**) Pulmonary findings related to HF in CT. A: Bilateral pleural effusion; B: Ground-glass opacities in gravity-dependent part of the lungs; C: Smooth septal thickening. (**right**) Image of the lungs in HF. A: Bilateral pleural effusion; B: interlobar septal thickening; C: Thickening of interlobular fissures; D: Ground-glass opacities in gravity-dependent part of the lungs. Figure obtained with permission from the 2nd Department of Radiology, Medical University of Warsaw.

**Figure 11:**
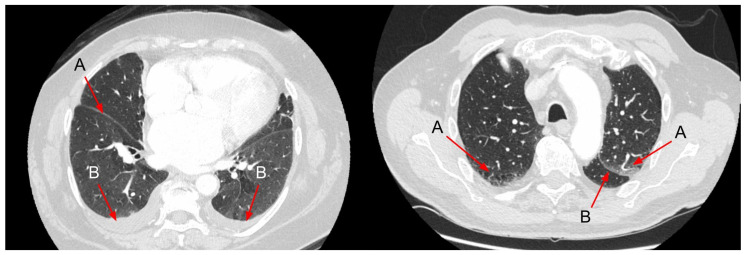
(**left**) Image of the lungs in CT in a patient with HF. Note the non-homogenous increased diffuse parenchymal density of the lungs. A: Thickening of interlobar fissure; B: Bilateral pleural effusion. (**right**) A: Cephalisation—widened upper lobe vasculature. B: Thickening of interlobar fissure and marginally increased density of the interstitium with a patchy appearance in the gravity-dependent part of the lungs within the lobes. Figure obtained with permission from the 2nd Department of Radiology, Medical University of Warsaw.

The most common features of hydrostatic oedema in the lungs are ground-glass opacities, interlobular septal thickening, peribronchovascular interstitial thickening, diffuse heterogeneous increase in lung density, pleural effusion, consolidation and mediastinal lymphadenopathy [71,72,73,74,75] (Figure 10 and Figure 11). Among them, interlobular septal thickening and pleural effusion proved to be the best predictors of HF independent of renal insufficiency [76].

It is challenging to compare the lung changes, particularly interlobular septal thickening representing lymphatic oedema and centrally located ground-glass opacities arising in the course of HF and interstitial pulmonary diseases. The main differences in patterns of interstitial changes are listed in Table 1 [77,78]. Recent studies emphasise the significance of early identification of tiny interstitial lung abnormalities preceding the diagnosis of ILD. A similar diagnostic dilemma refers to interstitial pneumonia with autoimmune features (IPAF), in which the presence of lung changes precedes the final diagnosis [78,79].

As experimental studies have documented, the features of HF usually follow a predictable sequence, beginning with interstitial oedema, while alveolar oedema and pleural effusion appear when the clearance mechanism in the interstitial space is overwhelmed by the accumulation of fluid [72,80]. Interstitial oedema is usually represented by smooth interlobular septal thickening and peribronchovascular thickening. Rarely, CHF can manifest as increased diffuse parenchymal density reflected by subtle changes in Hounsfield units [81]. Increased density represents diffuse alveolar oedema and thus may explain why pleural effusion may be seen without other manifestations of alveolar oedema. In some patients, ill-defined perivascular and centrilobular opacities or ground-glass opacity may appear with a tendency to have a parahilar and gravitational distribution [21].

**Figure 12:**
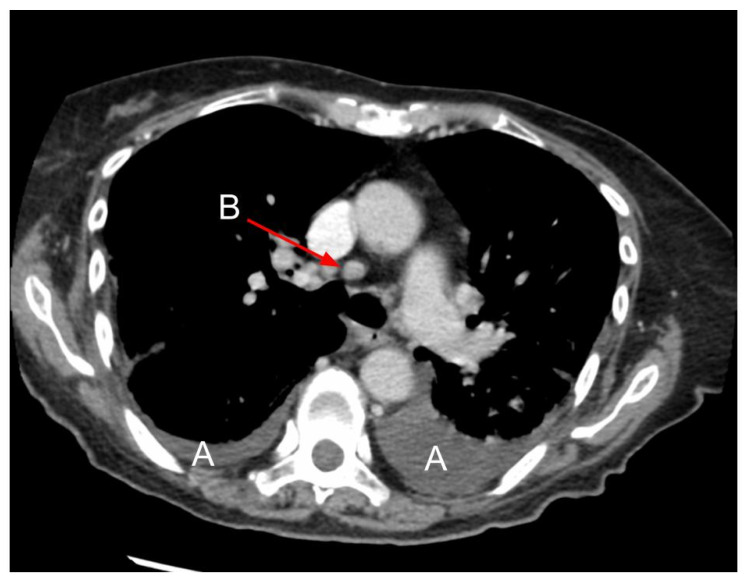
Mediastinal lymphadenopathy in CT. A: Bilateral pleural effusion; B: Lymphadenopathy of pretracheal lymph nodes (4R). Figure obtained with permission from the 2nd Department of Radiology, Medical University of Warsaw.

**Figure 13:**
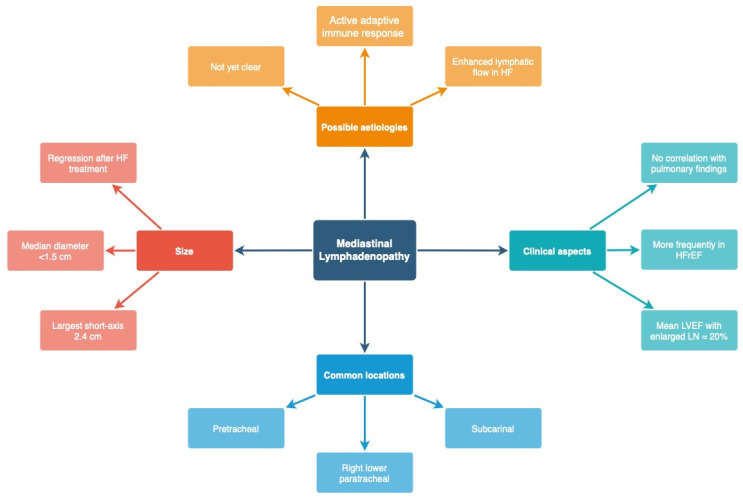
Characteristics of mediastinal lymphadenopathy related to heart failure. HFrEF—Heart failure with reduced ejection fraction; LN—lymph nodes; HF—heart failure.

Enlarged mediastinal lymph nodes are not a common feature of HF and do not correlate with the presence of interstitial findings typical for pulmonary oedema or the presence of pleural effusion [21,74,82]. Mediastinal lymphadenopathy is present mostly in patients with HFrEF. Most frequently, pretracheal (4R), right lower paratracheal (4R) and subcarinal lymph nodes are slightly enlarged, as their median diameter is usually below 1.5 cm [83] (Figure 12). Regression of the enlarged lymph nodes was documented after successful treatment of HF [83]. The pathomechanism of mediastinal lymph node enlargement is not clear. Sintou et al. documented an active adaptive immune response against the heart with the presence of anti-heart autoantibodies in the serum as well as deposited in the myocardium in both rats with experimental HF and human HF patients [84] (Figure 13).

The summarising table of main findings, advantages and disadvantages of all three above-mentioned imaging techniques can be found below (Table 2). Among other imaging tests useful in the diagnostic work-up of patients with HF, echocardiography, cardiac magnetic resonance, single-photon emission CT (SPECT) and computed tomography coronary angiography are the most common. However, they are aimed at cardiac assessment, and lung abnormalities are rarely detected by them.

## 5. Conclusions

In conclusion, the spectrum of lung changes related to HF diagnosed in chest imaging is wide, thus leading to a diagnostic dilemma. The precise knowledge of different types of these abnormalities allows us to distinguish patients with HF from patients with lung diseases or conditions in which both (lung and heart) are affected and thus avoid unnecessary diagnostics or therapies. In light of recent studies, LUS emerged as the most sensitive, repeatable and safe tool for both the diagnosis and management of HF. At the same time, both CXR and CT are more appropriate for the differential diagnosis in patients with suspicion of HF.

## Figures and Tables

**Table 1:** Comparison of HF- and ILD-related changes in CT lung image.

HF-Related	ILD-Related
Interlobar septal thickening (smooth)	Interlobar septal thickening (smooth or beaded)
Mediastinal lymphadenopathy	Mediastinal lymphadenopathy
Diffuse heterogeneous increase in lung density	Mosaic attenuation
Ground-glass opacities	Ground-glass opacities
Consolidation	Consolidation
	Reticular/reticulonodular pattern Tree-in-bud sign Subpleural curvilinear line Honeycombing Traction bronchiectasis, bronchiolectasis

**Table 2:** Main findings in different stages of the pulmonary image in HF. A comparison of the advantages and disadvantages of various radiographic modalities is presented.

		CXR	LUS	CT
**Main findings**	Redistribution	- Cephalisation - Broaden vascular pedicle	- No distinctive changes	- Enlarged vascular diameter - Cephalisation (within lobes)
	Interstitial pulmonary oedema	- Distorted hilar image - Peribronchovascular cuffing - Kerley lines	- The presence of >2 B-lines in one intercostal space - Homogeneous, bilateral, gravity-dependent distribution	- Interlobular septal thickening - Peribronchovascular thickening - Mediastinal lymphadenopathy
	Alveolar pulmonary oedema	- Angel wing/bat wing/butterfly pattern	- Increasing number of B-lines - Interstitial alveolar syndrome (sonographic “white lung”)	- Increased density - Ground-glass opacity - Consolidation
	Pleural effusion	- Detectable > 200 mL (PA) and > 100 mL (lateral) - Poor accuracy in determining the fluid character	- High sensitivity—detection > 50 mL (the most sensitive imaging) - Additional information about the fluid character	- High sensitivity - Limited accuracy in determining fluid character
**Advantages**		- Moderate accessibility - Inexpensive	- High accessibility (quick bedside examination) - Inexpensive - Higher sensitivity than CXR - Following the actual clinical response to therapy - No radiation dose - Easily repeatable	- Differential diagnosis - High sensitivity - The highest resolution
**Disadvantages**		- Limited sensitivity - Gravity-dependent - High interobserver variability - Minor radiation exposure (PA 0.1 mSv;)	- Limited surface/visualisation - Patient-dependent (low- quality study in the obese) - Training required	- Scarce availability - Expensive - High radiation risk (LD-CT 1–2 mSv; standard CT 7–8 mSv)

## Data Availability

No new data were created or analyzed in this study. Data sharing is not applicable to this article.

## Abbreviations

AHF	Acute heart failure
BNP	B-type natriuretic peptide
CHF	Chronic heart failure
CT	Computed tomography
CTR	Cardiac thoracic ratio
CXR	Chest X-ray
ECHO	Echocardiography
ED	Emergency Department
EF	Ejection fraction
EVLW	Extravascular lung water
HF	Heart failure
HFmrEF	Heart failure with mid-range ejection fraction
HFpEF	Heart failure with preserved ejection fraction
HFrEF	Heart failure with reduced ejection fraction
ICU	Intensive Care Unit
ILD	Interstitial Lung Disease
LUS	Lung ultrasound
LV	Left ventricle
NSTEMI	Non-ST elevated myocardial infarction
STEMI	ST elevated myocardial infarction
